# Passive immunization for influenza through antibody therapies, a review of the pipeline, challenges and potential applications

**DOI:** 10.1016/j.vaccine.2016.08.057

**Published:** 2016-10-26

**Authors:** Erin Sparrow, Martin Friede, Mohamud Sheikh, Siranda Torvaldsen, Anthony T. Newall

**Affiliations:** a The World Health Organization, Geneva, Switzerland; b School of Public Health and Community Medicine, University of New South Wales, NSW, Australia; c Sydney Medical School Northern, University of Sydney, NSW, Australia

**Keywords:** Influenza, Monoclonal antibody, Immunization, Pandemic, Vaccine, Prophylaxis, bNAb, broadly neutralizing antibody, GAP, Global Action Plan for influenza vaccines, mAb, monoclonal antibody, ICTRP, International Clinical Trials Registry Platform

## Abstract

The Global Action Plan for influenza vaccines (GAP) aims to increase the production capacity of vaccines so that in the event of a pandemic there is an adequate supply to meet global needs. However, it has been estimated that even in the best case scenario there would be a considerable delay of at least five to six months for the first supplies of vaccine to become available after the isolation of the strain and availability of the candidate vaccine virus to vaccine manufacturers. By this time, the virus is likely to have already infected millions of people worldwide, causing significant mortality, morbidity and economic loss.

Passive immunization through broadly neutralizing antibodies which bind to multiple, structurally diverse strains of influenza could be a promising solution to address the immediate health threat of an influenza pandemic while vaccines are being developed. These products may also have a role in seasonal influenza as an alternative to other options such as antivirals for the treatment of severe acute respiratory illness due to influenza.

This article provides an overview of the current clinical pipeline of anti-influenza antibodies and discusses potential uses and the challenges to product development.

## 1 Introduction

Both influenza A and influenza B viruses cause seasonal epidemics in humans. Seasonal influenza vaccines contain attenuated strains of influenza A and influenza B viruses. The subtypes contained within seasonal influenza vaccines can vary from year to year due to minor changes in the genetic makeup of the viruses known as antigenic drift. Antigenic drift occurs on a continuous basis as the influenza virus replicates and is the reason why lifelong immunity does not occur following natural infection. Twice per year the World Health Organization (WHO) issues recommendations on the composition of seasonal influenza vaccines for the northern and southern hemispheres. Influenza A viruses also have the potential to undergo major genetic changes, known as genetic shift, which can cause pandemics.

In order to mitigate the spread and severity of an influenza pandemic multiple strategies are needed. Vaccines may remain one of the best defences against a pandemic, however the need for the vaccine to be made specifically to the pandemic strain, and the time needed for vaccine production means that there is a delay of several months before vaccines would be available to the general population [1]. It is likely that by this time the virus will have spread to infect millions of people worldwide bringing with it significant mortality and economic loss [2], [3]. Such a delay in vaccine availability was experienced during the 2009 pandemic (A(H1N1)pdm09) with the virus identified in April and Candidate Vaccine Virus available to manufacturers in May, but the first vaccines not ready for distribution until October that year [4]. One strategy undertaken by several Governments is to stockpile “pre-pandemic” vaccines against avian subtypes such as H5N1 or H7N9. However, there are uncertainties about what the next pandemic strain will be and whether stockpiled vaccines would be efficacious against it [5], [6]. While “universal vaccines” that could protect against any influenza strain would avoid this delay in vaccine availability, such products are still very far from reality. A further limitation is that with active immunization with vaccines, there would also be a delay of about two weeks between immunization and development of protective immunity.

The Global Action Plan for Influenza Vaccines (GAP) was created to address global concerns about access to pandemic influenza vaccines. The third objective of GAP was to promote research and development of improved influenza vaccines. Since then, some progress has been made in the development of novel influenza vaccines and production technologies and there are several innovative vaccines approved or in development [7], [8]. A truly “universal” influenza vaccine would ideally confer lifelong immunity for all influenza subtypes and be unaffected by antigenic drift and shit. However, major barriers to the development of “universal” influenza vaccines are: a lack of consensus on the primary clinical endpoint to be achieved; a lack of correlates of protection to measure success; and the extensive and costly efficacy trials anticipated [7]. Taking into consideration the challenges to development of universal influenza vaccines, the WHO Product Development for Vaccines Advisory Committee has considered the improvement of seasonal influenza vaccines to be more feasible and advised WHO to develop preferred product characteristics for seasonal vaccines to improve the breadth, quality and duration of protection [9].

During the last decade there has also been extensive research on monoclonal antibodies for passive immunization against influenza. Such products could be used as pre- or post-exposure prophylaxis to prevent or reduce symptoms or in the treatment of severe influenza infection.

Passive immunization with recombinant antibodies presents an alternative strategy that could be implemented early in the pandemic to mitigate the impact of the virus while vaccines are being manufactured. If such products were broad spectrum and targeted conserved regions of the influenza A virus their efficacy may not be affected by antigenic drift or shift, meaning that the same products could be used from year to year in seasonal epidemics and be stockpiled for use in pandemics.

There are several advantages of monoclonal antibodies over vaccines including a potentially easier and more feasible research and development (R&D) pathway than universal vaccines, and a rapid onset of protective immunity. However, these advantages have to be contrasted with a higher cost, limited production capacity and the relatively short duration of protection.

In addition to bridging the gap between the start of the pandemic and vaccine availability, recombinant antibodies may also offer a promising alternative to other antiviral treatment options such as oseltamivir, which have shown limited efficacy in treating patients with influenza [10]. Furthermore, such products may also be a potential therapy for severe influenza caused by circulating seasonal strains of influenza A. Their use as a treatment during seasonal epidemics would ensure an annual market for manufacturers, leading to the establishment of facilities and sustainable production lines to be called upon in the event of a pandemic.

After ten years the GAP is coming to a close and a consultation with global stakeholders will mark its end in November 2016. When reviewing the progress under objective three of the GAP, stakeholders should look beyond progress made in vaccine development to consider also the development of recombinant antibodies for passive immunization against influenza.

Here we review the current pipeline of recombinant anti-influenza antibodies in clinical development, discuss some of the challenges to their product development, licensure and use, and discuss their public health potential and potential cost-effectiveness. The article also presents unresolved questions for developers and regulatory authorities to consider.

## 2 Methods

To determine the current pipeline of influenza antibodies in clinical development, a review of Clinical Trials.gov and of the WHO International Clinical Trials Registry Platform (ICTRP) was conducted on 8 January 2016 using the keywords “Monoclonal AND Influenza”. The search yielded clinical trials for eight different monoclonal antibody products. To obtain more information regarding preclinical and clinical trial results, mode of action, dosing, efficacy and target product profiles literature and web reviews were conducted. To obtain published articles in scientific journals a literature review in PubMed using the candidate drug name was conducted. To obtain grey literature such as abstracts from conferences and press releases a review of manufacture’s websites, Google Scholar and Google was then conducted for each of these candidate drugs.

## 3 Results

We identified eight different monoclonal antibody candidate products registered to be currently or have been in clinical development (Table 1). All products have undergone in vitro testing and in vivo evaluation in animal models. No products have made it beyond the phase 2 clinical trial stage. All products are expressed through mammalian cell lines and administered intravenously. All products are targeted against influenza A and reported to be broad spectrum across various subtypes of the virus. The majority of products are being developed by companies that are US based or owned. A summary of the information on the state of development for each of the eight products found in the public domain is presented below in alphabetical order.

### 3.1 CR6261

Crucell’s candidate mAb CR6261 is directed against a highly conserved helical region in the membrane-proximal stem of hemagglutinin. In vitro studies on this class of antibodies have demonstrated neutralization activity across a broad spectrum of influenza A subtypes. Crucell’s CR6261 mAb has been shown to be protective in mice studies against lethal doses of H1N1 and H5N1 viruses. A challenge study has also been conducted to successfully demonstrate both therapeutic and prophylactic efficacy in the ferret model against a lethal dose of H5N1 (A/Indonesia/5/2005) [11]. According to the clinical trial’s registration, phase 2 of Crucell’s CR6261 (NCT02371668) is still undergoing recruitment and is estimated to be completed in May 2017. The trial is designed to evaluate CR6261 compared to placebo 24 h after challenge with H1N1 virus. The selected dose being evaluated is 50 mg/kg administered intravenously as a single dose over two hours.

### 3.2 CR8020

Crucell’s other candidate mAb, CR8020, targets an immune-subdominant, relatively conserved membrane-proximal stem region of hemagglutinin. The mAb specifically recognizes group 2 influenza viruses and has been shown to have neutralization activity against H3, H7 and H10 subtypes [12], [13]. Studies in mice have shown the mAb to have both prophylactic and therapeutic efficacy against H3N2 and H7N7 viruses [14]. A phase 2a trial of the mAb was completed in 2014 (NCT01938352/EUCTR2013-002185-39-GB). The trial was designed to evaluate the prophylactic effects of CR8020. Published results were not identified, but some results have been entered in the EU Clinical Trials Register [15]. A total of 22 subjects received the product (15 mg/kg) or placebo 2 days after inoculation with an influenza challenge virus. The primary endpoint was area under the curve of viral load. Secondary endpoints were: percentage of participants with detected and quantitative influenza infection rate; area under the curve influenza symptoms; antibody serum concentration.

### 3.3 CT-P27

Celltrion’s candidate, CT-P27 is a dual antibody product containing two monoclonal antibodies (CT-P22 and CT-P23) that are complimentary against a broad range of influenza A subtypes. Celltrion reports that their product has shown efficacy in in vitro neutralization studies and in vivo animal studies across various subtypes (H1N1, H2N2, H5N1, H9N2, H3N2, H7N9) [16]. The candidate has successfully been evaluated in phase 1 clinical trials [16] and is currently being evaluated in a phase 2a study (NCT02071914/EUCTR2013-004544-32-GB). The phase I study (KCT0001617) evaluated the safety and pharmacokinetics of CT-P27 administered intravenously both with and without oseltamivir at doses ranging from 40 mg/kg to 90 mg/kg. The phase 2a will evaluate CT-P27 in an influenza challenge model with H3N2 influenza, detailed information regarding the dosing to be evaluated is not available.

### 3.4 FGI-101-1A6

Functional Genetics Inc. had registered a phase 1 trial of their candidate mAb FGI-101-1A6 in 2011 (NCT01299142). The mAb is not directed against influenza directly but targets a protein on the surface of cells infected with certain viruses including influenza. According to a press release issued in November 2011, the phase 1 trial had been successfully completed [17]. However the functional genetics website is no longer active and further information could not be identified, therefore product development is assumed to have been suspended.

### 3.5 MEDI8852

MedImmune’s MEDI8852 targets a region within the stalk of the hemagglutinin protein that is highly conserved amongst all influenza A subtypes and is reported to bind to all 18 subtypes. The mAb has been evaluated and shown to be efficacious in a lethal seasonal influenza mouse challenge compared to and in combination with oseltamivir. A similar study in ferrets was conducted with animals challenged with H5N1 virus. In the ferret study, treatment with the mAb was more efficacious than the antiviral in preventing death, lowering temperature, and reducing the amount of clinical symptoms. MEDI8852 alone was more protective than oseltamivir alone and the combined group provided the greatest protection [18]. The product has also entered clinical trials with a phase 1 completed (NCT02350751) and a phase 1b/2a (NCT02603952) with expected completion in May 2016. The phase 1b/2a will evaluate a single dose of MEDI8852 when co-administered with oseltamivir compared with MEDI8852 alone and oseltamivir alone in adult subjects with acute, uncomplicated influenza. No clinical trial results have been published. The drug recently received Fast Track designation from the US FDA which should accelerate its development [19].

### 3.6 MHAA4549A

Genentech’s candidate mAb MHAA4549A targets a highly conserved epitope on the stalk region of hemagglutinin in both group 1 and group 2 influenza A viruses. The mAb has been show to neutralize all seasonal influenza A viruses [20]. It has also been evaluated and shown to be effective in mice and ferret challenge studies against H5N1 virus [21]. The product has also successfully completed phase 1 clinical trials and is currently being evaluated in phase 2. One phase 2a study has been completed (NCT01980966). This trial was designed to compare the efficacy and safety of MHAA4549A in healthy volunteers challenged with influenza virus. No clinical trial results were identified. Other phase 2 studies are currently recruiting. NCT02293863/EUCTR2014-000461-43 will compare MHAA4549A in combination with oseltamivir compared to oseltamivir alone in hospitalized patients with severe influenza A and is expected to be completed in June 2017. NCT02623322 will evaluate the candidate mAb against placebo in otherwise healthy adults with acute uncomplicated seasonal influenza A and is expected to be completed in July 2017.

### 3.7 TCN-032

Theraclone’s candidate TCN-032 has completed a phase 2a clinical trial. The candidate targets an epitope which is present in >99.8% of reported human, avian and swine influenza A strains [22]. The monoclonal antibody was evaluated in mouse challenge studies and showed survival benefit against lethal challenge with both H5N1 (A/Vietnam/1203/04) and H1N1 influenza (A/Puerto Rico 8/34) [23]. A phase 1 study evaluated the mAb given intravenously at varying single doses ranging from 1 to 40 mg/kg for safety and pharmacokinetics. Published results were available for the phase 2a trial (NCT01719874/EUCTR2012-000854-73-GB) [22]. The trial was conducted on healthy volunteers who were challenged with H3N2 influenza and administered intravenously either the mAb at a single dose of 40 mg/kg or placebo 24 hours later. The drug was shown to be safe however failed to meet its primary endpoint which was to reduce the proportion of subjects developing grade ⩾2 influenza symptoms or pyrexia during seven days post viral challenge. The product did achieve some secondary objectives such as a reduction in viral shedding and was shown to significantly reduce influenza symptoms including duration when compared to the placebo. Reduction in symptoms observed in this study were similar to reported results in other human challenge models with oseltamivir and peramivir. These results suggest that the product may provide therapeutic benefit for the treatment of influenza.

### 3.8 VIS410

Visterra’s candidate drug VIS410 targets a unique, highly conserved and constrained epitope on hemaglutinin. The product has been tested in vitro and shown to neutralize over 40 different strains. Mouse challenge studies have been conducted showing protection against various subtypes of influenza including strains of H1N1, H3N2, H5N1 and H7N9. Ferret studies have also been conducted with H1N1 demonstrating that VIS410 may also prevent aerosol transmission of H1N1 influenza [24]. The candidate has also been evaluated in phase 1 and phase 2 clinical trials. Published results were available for the phase 1 trial (NCT02045472) [25]. The study was conducted on healthy volunteers at dose levels ranging from 2 to 50 mg/kg. The trial was primarily a safety study which also looked at pharmacokinetics in both serum and the upper respiratory tract. Safety and PK results paired with animal data suggest this product may provide therapeutic benefit. A phase 2a study has been completed (NCT02468115) to assess the effect of VIS410 in healthy subjects 24 hours after inoculation with H1N1 with a single (undefined) dose of VIS410. Full results are not available but the study was reported on Visterra’s website to have been successful in reaching its primary endpoint (area under the curve of viral shedding over time). The developer’s website also reports to be planning to conduct a Phase 2a clinical trial in ambulatory patients with confirmed influenza and a Phase 2b clinical trial in hospitalized patients with influenza in 2016 [26].

## 4 Discussion

With the exception of antibodies against Respiratory Syncytial Virus and anthrax, the majority of monoclonal antibodies on the market today are for the treatment of chronic conditions. Due to their complex nature, monoclonal antibodies are a class of product subject to specific regulatory requirements, and many regulatory agencies have published guidance for development of mAbs. Discussed below are challenges specific for product development of influenza mAbs rather than regulatory considerations for mAbs in general.

Several promising anti-influenza monoclonal antibody products are in development and may have a benefit in the pre- or post-exposure prophylaxis or treatment of both seasonal and pandemic influenza A infection.

In addition to these candidates in clinical trials described here, there are numerous novel candidates being developed that are still in the preclinical phase. Many of these candidates have been demonstrated to have protective and therapeutic efficacy in animal challenge models and may soon enter clinical trials in humans.

There are however challenges to the development of these products and in addition to the current lack of data regarding their efficacy in humans, there are several unknowns regarding their appropriateness for widespread use and potential cost-effectiveness.

While in vitro neutralization data and in vivo animal studies have demonstrated prophylactic and therapeutic potential, efficacy studies are needed for all candidates to demonstrate utility in humans for pre- and post-exposure prophylaxis or as a treatment in humans infected with influenza. Such studies should be conducted against multiple strains of influenza where possible.

Human challenge studies can be useful to demonstrate pharmacological antiviral activity, however, according to the US FDA “Guidance for Industry, Influenza: Developing Drugs for Treatment and/or Prophylaxis” challenge studies should not be used to demonstrate efficacy as the challenge strains used are less virulent and produce milder symptoms than natural influenza infection [27], [28].

For demonstrating therapeutic efficacy, studies in humans infected with severe influenza can be conducted. This can certainly be demonstrated for circulating seasonal influenza A strains (H1N1, H3N2). However this becomes more difficult for non-circulating strains. Demonstrating efficacy to non-seasonal strains of influenza such as avian H7N9 or H5N1 is extremely challenging due to their sporadic and low incidence in humans and their occurrence in only a few countries. For such targets, animal challenge data paired with safety data and efficacy data in humans for seasonal influenza strains will need to be relied on for licensure by regulatory authorities.

With regards to demonstrating efficacy in clinical trials, clinical endpoints need to be well-defined. The primary outcome measure leading to licensure needs to be agreed in advance with regulatory agencies. For the case of prophylaxis, US FDA guidance specifies that “the primary endpoint for trials aiming to demonstrate efficacy as prophylaxis should be the occurrence of symptomatic, laboratory-confirmed influenza” [28].

For the case of treatment of hospitalized patients, US FDA guidance states that the primary endpoint should include “clinical signs and symptoms, duration of hospitalization, time to normalization of vital signs and oxygenation, requirements for supplemental oxygen or assisted ventilation, and mortality” [28]. As a clinical endpoint this definition is rather broad and needs to be better defined. Furthermore it would be quite difficult to measure a statistically significant reduction in mortality, especially in highly industrialized countries where treatment of patients hospitalized with influenza is generally successful. Powering such studies would require very large sample sizes and therefore high costs to implement.

If possible, it is important for developers to demonstrate both prophylaxis and treatment benefits as there may very well be benefit in a prophylactic product that has minimal impact on reducing symptoms but that prevents the infection leading to hospitalization.

While trials in healthy humans can provide evidence of efficacy, trials should also be conducted on at risk-populations. Populations at a higher risk of influenza infection include such groups as pregnant women, the elderly, children or persons with underlying chronic conditions especially the immunocompromised. These populations are most at risk of severe influenza infection and therefore drugs are more likely to be used in these groups, so demonstrating efficacy and safety in these populations is important as they may not respond the same as healthy adults.

It is particularly important that trials are designed well and in discussion with regulatory agencies in order to demonstrate true benefits of mAbs for prophylaxis or treatment of influenza. The therapeutic efficacy of two antiviral drugs (oseltamivir and zanamivir) which are recommended for use in treating and preventing both seasonal and pandemic influenza has been called into question following a Cochrane Review published in January 2012 and updated in April 2014 [29], [30]. The review, which reviewed both published and unpublished data from clinical trials, concluded that while both drugs had prophylactic efficacy, their therapeutic efficacy was modest in alleviating symptoms and that data was insufficient to demonstrate whether they reduce complications of influenza such as pneumonia or bronchitis.

Given that the conclusions drawn from the Cochrane review came from published and unpublished trials, it is important that developers are transparent in making all results of all of their trials publically available. The WHO calls for all clinical trial results to be published, including negative results [31].

Some concern has also been raised about the potential of influenza mAbs to cause antibody-dependent enhancement of illness as has been observed for Dengue virus [32]. Such an immunological outcome must also be carefully evaluated in clinical trials.

A major question with regards to the use of such products is their potential cost effectiveness. The current estimated cost to produce one gram of monoclonal antibody is US$ 100/gram [33]. Using an example of a 70 kg adult, requiring 40 mg per kg (the dose being evaluated for TCN-032 in a post-exposure prophylaxis human challenge study) 2.8 g would be required. In this example, the estimated cost to manufacture this dose would be approximately $US 280. The sales price may be substantially higher than this, but even at the current cost of production alone this is more expensive than existing small molecule antivirals. Therefore, at current production costs when compared to antiviral use for post-exposure or treatment, the efficacy of mAbs would likely have to be substantially greater than small molecule antivirals to present a cost-effective strategy. However, other developers of candidate influenza mAbs have not made their selected dosing publically available and have evaluated doses as low as 1 mg/kg in their phase 1 studies. If such low does are efficacious they may be able to be priced at a lower cost and consequently be more likely to be cost-effective to use.

In terms of pre-exposure prophylaxis prevention, the cost of mAbs use is likely to be substantially higher than the cost of influenza vaccines. Therefore, in situations where influenza vaccines can be used, and be the comparator in an economic evaluation, mAbs are unlikely to be cost-effective. This is similar to the current situation with antivirals use for prevention of seasonal influenza when compared to influenza vaccine. The use of mAbs for prevention may potentially be more suitable in cases where an effective influenza vaccine cannot be used. For example, in situations where it is not immediately available (e.g. in a pandemic scenario), when an influenza vaccine cannot be provided to the individual (e.g. in children under 6 months), or when it will not offer protection in time to prevent infection (e.g. after exposure to an infected individual). In these cases, the relative costs and benefits of mAbs use for prevention will need to be compared to other interventions that can potentially be used in this situation. The duration of protection for a given dose of mAb may be influential in determining cost-effectiveness in the context of prevention.

For potential use as prophylaxis or treatment in a pandemic situation such products could be stockpiled. However, their shelf-life and storage conditions must be conducive to stockpiling. Storage at −80 °C for instance would not be ideal. The time to the next pandemic is unknown but may be many years away, so a long shelf-life would also be required, with demonstrated stability over multiple years. Developers should ensure that this is measured during product development. The cost-effectiveness of interventions against pandemic influenza is difficult to estimate as there is substantial uncertainty in key factors such as when the next pandemic will occur and how severe it will be. However, governments in many countries have been willing to stockpile antivirals and other measures for use in a future pandemic. If mAbs are approved, decision makers wishing to stockpile pharmaceuticals for a pandemic should consider the relative merits of mAbs in the context of other options available to them.

A final challenge to this class of product is that all candidates reviewed are currently being evaluated as a single dose administered intravenously over several hours. Such administration would really only be applicable in a well-resourced hospital setting and would limit their use for prophylaxis and for use in developing countries. It would also add to the cost of treatment, especially if several doses were required. Alternative forms of administrations such as intramuscular injection would overcome this challenge. Low doses could potentially be administered by intramuscular injection, but large gram-quantity doses would need to be administered intravenously over several hours.

## 5 Conclusion

There is a need for products to prevent or treat influenza early in a pandemic while vaccine manufacturing is occurring. Influenza monoclonal antibodies are a class of product which could help address this need but further clinical trials are needed to demonstrate their efficacy.

While influenza mAbs may be shown to be efficacious, it is doubtful that such products would have a wide-spread use in a pandemic especially in developing countries due to their anticipated high cost and current difficult administration. However, they could certainly have a targeted use in the prophylaxis of certain at risk populations such as pregnant women and other risk groups and for the treatment of hospitalized patients with severe influenza infection. To minimize the barriers of cost, supply and administration, products which could be administered by simple intramuscular injection should be prioritised. Such products would probably need to be very high affinity, and for prophylactic use ideally with an extended half-life similar to the product being developed by Medimmune for RSV [34].

As monoclonal antibodies are more expensive to produce than chemically synthesised antiviral drugs, the cost effectiveness of monoclonal antibodies compared to small molecule antivirals for influenza must also be established once their efficacy has been demonstrated and pricing is known. Such information will be important for governments and insurers in deciding on reimbursement/subsidization of such products.

If shown to be efficacious and cost effective, monoclonal antibodies currently in clinical development would certainly have a use in treating hospitalized patients for seasonal influenza A in countries with well-developed health systems and willingness to pay the associated costs. They may also be considered by governments for stockpiling for use in a pandemic situation.

## WHO disclaimer

The authors alone are responsible for the views expressed in this article, which do not necessarily represent the views, decisions or policies of the institutions with which the authors are affiliated.

## Conflict of interests

No authors have any conflict of interest.

## Funding

This research did not receive any specific grant from funding agencies in the public, commercial, or not-for-profit sectors.

## Figures and Tables

**Table 1:** Pipeline of monoclonal antibody products for influenza under clinical development.

Candidate name	Manufacturer/developer	Preclinical studies	Latest clinical phase and status	Clinical trial registration numbers	Mode of action	Route of administration and dose	Clinical trial results
CR6261	Crucell	In vitro neutralization, in vivo mice and ferret challenge studies	Phase 2 recruiting	Phase 1: NCT01406418	Directed against a highly conserved helical region in the membrane-proximal stem of hemagglutinin	Single dose, intravenous infusion, 50 mg/kg	No clinical trial results available
				Phase 2: NCT02371668			
CR8020	Crucell	In vitro neutralization and in vivo mice challenges	Phase 2a completed	Phase 1: NCT01756950	Directed against an immune-subdominant, relatively conserved membrane-proximal stem region of hemagglutinin	Single dose, intravenous infusion, 15 mg/kg	Some clinical results available for NCT01938352/EUCTR2013-002185-39-GB [11]
				Phase 2a: NCT01938352 and EUCTR2013-002185-39-GB			
CR802 and CR6261 combined	Crucell	See above	Phase 2a withdrawn prior to enrolment	Phase 2a: NCT01992276 EUCTR2013-003341-41	As above	Single dose, intravenous infusion, 30 mg/kg	N/A (withdrawn)
CT-P27	Celltrion	In vitro neutralization, in vivo mice and ferret challenge studies	Phase 2a ongoing	Phase 1: KCT0001617	Dual antibody product. Antibodies bind a conformational epitope on the hemagglutinin stem region	Single dose, intravenous infusion (dose not mentioned)	No clinical trial results available
				Phase 2a: NCT02071914and EUCTR2013-004544-32-GB			
FGI-101-1A6	Functional Genetics Inc.	N/A	Phase 1 status unknown	Phase 1: NCT01299142	Targets the TSG101 protein on the surface of host cells infected by viruses	Single dose, intravenous infusion (dose not mentioned)	Press release available [13]
MEDI8852	MedImmune	In vitro neutralization, in vivo mice and ferret challenge studies	Phase 1b/2a recruiting	Phase 1: NCT02350751	Binds to a novel site in the hemagglutinin stem region that is shared in viruses from all 18 Influenza A subtypes	Single dose, intravenous infusion (dose not mentioned)	No clinical trial results available
				Phase 1b/2a: NCT02603952			
MHAA4549A	Genentech	In vitro neutralization, in vivo mice and ferret challenge studies	1 phase 2a trial completed (NCT01980966).	Phase 1: NCT02284607	Binds to a highly conserved epitope on the stalk of influenza A hemagglutinin	Single dose, intravenous infusion (dose not mentioned)	No clinical trial results available
				Phase 1: NCT01877785			
			Other phase 2 trials recruiting	Phase 2a: NCT01980966			
				Phase 2: NCT02293863 and EUCTR2014-000461-43			
				Phase 2: NCT02623322			
TCN-032	Theraclone Sciences	In vitro neutralization and in vivo mice challenges	Phase 2a completed	Phase 1: NCT01390025	Directed against conserved epitope of the amino-terminal extracellular domain (M2e) of the influenza virus matrix protein 2 (M2)	Single dose, intravenous infusion, dosing from 1 to 40 mg/kg evaluated	Results published for NCT01719874 [18]
				Phase 2a: NCT01719874 and EUCTR2012-000854-73-GB			
VIS410	Visterra Inc.	In vitro neutralization, in vivo mice challenges and ferret studies	Phase 2a completed	Phase 1: NCT02045472	Directed against a hierotope on hemagglutinin	Single dose, intravenous infusion (dose not mentioned for phase 2)	Results published for NCT02045472 [21]
				Phase 2a: NCT02468115			
